# Hybridization and introgression of native and foreign *Sorbus* tree species in unique environments of protected mountainous areas

**DOI:** 10.1093/aobpla/plaa070

**Published:** 2020-12-30

**Authors:** Anna Hebda, Marta Kempf, Witold Wachowiak, Bartosz Pluciński, Paweł Kauzal, Tomasz Zwijacz-Kozica

**Affiliations:** 1 Department of Plant Biotechnology, Faculty of Biochemistry, Biophysics and Biotechnology, Jagiellonian University in Kraków, Kraków, Poland; 2 Department of Genetics and Forest Tree Breeding, Institute of Forest Ecology and Silviculture, Faculty of Forestry, University of Agriculture in Krakow, Kraków, Poland; 3 Institute of Dendrology, Polish Academy of Sciences, Kórnik, Poland; 4 Department of Plant Physiology and Biochemistry, Faculty of Biochemistry, Biophysics and Biotechnology, Jagiellonian University in Kraków, Kraków, Poland; 5 Tatra National Park, Zakopane, Poland

**Keywords:** Evolution, genetic diversity, hybridization, mating system, population structure, *Sorbus*

## Abstract

Hybridization and introgression are important processes influencing the genetic diversity and evolution of species. These processes are of particular importance in protected areas, where they can lead to the formation of hybrids between native and foreign species and may ultimately result in the loss of parental species from their natural range. Despite their importance, the contribution of hybridization and introgression to genetic diversity in *Sorbus* genus remains not fully recognized. We analysed the genetic and morphological variability of several *Sorbus* species including native (*Sorbus aria*), foreign (*S. intermedia*) and potentially hybrid (*S. carpatica*) individuals from the Polish Carpathian range. Patterns of variation at 13 nuclear microsatellite loci show hybridization between the tested species and confirm the existence of the hybrid form *S. carpatica*. Biometric analysis on leaves, based of 10 metric features and three parameters, identified several characters for preliminary taxonomic classification; however, none of them could be used as a fully diagnostic marker for faultless annotation of *S. intermedia* and *S. carpatica*. The genetic structure analysis indicated complex patterns of population differentiation and its diverse origin. The results allow assessment of genetic variation and identification of parental species participating in hybridization. This knowledge will advance the management of genetic diversity and development of conservation strategies for efficient maintenance of the unique protected ecosystem.

## Teaser

Hybridization, polyploidization and apomixis are important processes which affect the taxonomic complexity of *Sorbus* genus. The phenomenon of interspecific hybridization between native and foreign species is frequently a consequence of human activity. It may lead to increased diversity of plants but also it may significantly reduce genetic variation if invasive genotypes of natural species participate in the process. We report a preliminary study of the hybrid taxon occurring in natural and protected areas of Tatra National Park in Poland. Variation patterns at nuclear microsatellite loci were used to distinguish a hybrid form of *Sorbus carpatica* from morphologically very similar *S. intermedia*.

## Introduction

Hybridization is based on the transmission of alleles between species that may skew their frequency distribution in the population as expected under equilibrium either by the increase of polyploid levels or by introgression (Baack and Rieseberg 2007). The natural processes related to hybridization and polyploidization observed between plant species lead to their differentiation and adaptive evolution (Rieseberg *et al.* 2007; Doyle *et al.* 2008; Soltis and Soltis 2009; Ludwig *et al.* 2013; Hamston *et al.* 2018).

Newly formed polyploid hybrids constitute a separate group from parental taxa and they propagate most effectively by means of apomixis (Grant 1981; Mallet 2007). When apomixis is partial or facultative, backcrossing with parental individuals may occur (Richards 2003). Hybridization may lead to heterosis for growth (Bradshaw and Grattapaglia 1994) or survival ability. Moreover, it may produce endemics existing only in small populations unique to the local environment (Hamston *et al.* 2018). Hybrids characterized by improved quality and favoured by natural selection may ultimately displace parental species from their natural range (Rieseberg *et al.* 2007; Whitney *et al.* 2010; Andrew and Rieseberg 2013). Hybridization may also lead to outbreeding depression, when hybrids have reduced growth and survival (Chan *et al.* 2019). Because of the different outcomes of hybridization, it may be seen as a disturbing phenomenon and may pose a threat to the natural character of ecosystems, especially when hybrids spread without control (e.g. Wolfe *et al.* 2007; Chan *et al.* 2019). Therefore, hybridization between closely related species is of particular importance in protected ecosystems. Knowledge of genetic diversity of populations, gene flow and interbreeding may support conscious decisions in terms of gene resources conservation and management of protected areas.

The *Sorbus* genus is a good example to study the effects of hybridization, polyploidization and apomixis due to its taxonomic complexity that results from the combination of those processes (Ludwig *et al.* 2013). The genus includes about 250 species, which are distributed in the temperate zone of the northern hemisphere (Aldasoro *et al.* 1998). Only five of the European species are diploid and have a relatively wide range (Uhrinová *et al.* 2017). These are *Sorbus aria*, *S. aucuparia*, *S. torminalis*, *S. chamaemespilus* and *S. domestica*. The first four taxa are the main source of many hybrid and polyploid forms—endemic and stenobiotic taxa (Nelson-Jones *et al.* 2002; Gömöry and Krajmerová 2008; Pellicer *et al.* 2012). However, it is believed that the diploid *S. aria*, which can also form apomictic triploid and tetraploid species (Nelson-Jones *et al.* 2002; Lepší *et al.* 2015), is the main pollen donor that hybridizes with the other three diploid *Sorbus* taxa (Nelson-Jones *et al.* 2002; Gömöry and Krajmerová 2008). The morphological variability of *S. aria* is so high that it causes problems with distinguishing its polyploids from hybrids, without testing for polyploidy (Rich *et al.* 2010) or without other genetic analysis. Interspecies hybrids are well-recognized in the UK (Rich *et al.* 2010; Robertson *et al.* 2010; Ludwig *et al.* 2013). In Central- and East-Europe, in the area of the Western Carpathians, bigenomic hybrid species of *S. aria* and *S. chamaemespilus*, as well as trigenomic ones, involving also *S. aucuparia*, were found (Bernátová and Májovský 2003; Gömöry and Krajmerová 2008; Uhrinová *et al.* 2017). Earlier reports describe occurrences of hybrid forms belonging to *S. carpatica* in the Carpathians, which showed intermediate morphological features between *S. aria* and *S. austriaca* (Soó 1937 after Sennikov and Kurto 2017; Pawłowska and Pawłowski 1970) or *S. intermedia* (Giżycki 1845; Kárpáti 1960; Kutzelnigg 1994; Kovanda 1997). The exact distribution of *S. carpatica* in the Carpathians is not known. In Poland, it is classified as an endemic species in Tatra and Pieniny where *S. austriaca* is absent (Pawłowska and Pawłowski 1970). Several individuals of *S. carpatica* were identified in Slovakia, Hungary (Soó 1937 after Sennikov and Kurto 2017) and in the Czech Republic (Kovanda 1996) based on morphological analysis. However, subsequent analysis reclassified the plants as diploid *S. aria* with unusually lobed leaves (Lepší *et al.* 2015; Sennikov and Kurto 2017).

The interest in *Sorbus* species in the Tatra National Park (TNP), located in the Polish part of Carpathians originates mainly from the concerns about the genetic influence of foreign species, which may spread and interbreed with native species. Within the TNP, there are several stands of *S. intermedia* which were introduced artificially in the past or are a result of ornithochoria from trees located in the nearby urban greenery (Mirek 2016; Pusz *et al.* 2019). Interestingly, *S. intermedia* is a hybrid originating from *S. aria*, *S. aucuparia* and *S. torminalis* (Jankun 1993; Nelson-Jones *et al.* 2002; Robertson *et al.* 2010). Its natural distribution range is limited to southern Scandinavia and the Baltic coast, and the species has been cultivated in urban areas. It is known from Liljefors’ research (1954, 1955) that *S. intermedia* might have backcrossed with *S. aucuparia* or hybridized with *S. hybrida*. Hybrids resulting from backcrossing of *S. intermedia* with *S. aria* are very rare but were recognized in some parts of the world (Rich *et al.* 2010).

Considering the interspecific gene flow capacity of the *Sorbus* genus, we analysed the extent of hybridization and any potential genetic advantage that the hybrids may have over the native species within a unique area of TNP. We evaluated the so far unrecognized the variability and genetic structure of the two species: *S. aria* that is native to TNP, and the foreign *S. intermedia*. We studied variation of nuclear microsatellite DNA loci (e.g. Robertson *et al.* 2010; Hamston *et al.* 2018) and the variability of morphological features of leaves. In our work, we investigated if the tested *Sorbus* taxa are characterized by specific patterns of genetic diversity and structure that result from their hybridization and speciation in the presence of gene flow? Using our genetic data and leaf measurements, we aimed to establish what morphological diagnostic markers can allow faultless taxonomic classification of the studied *Sorbus* taxa. Obtained data provide an overview of *Sorbus* genetic resources of the unique area of TNP. We were also able to clarify the taxonomic position of *S. carpatica* and provide a more comprehensive overview of the evolutionary consequences of *Sorbus* hybridization.

## Materials and Methods

### Plant material and DNA extraction

**Figure 1.**
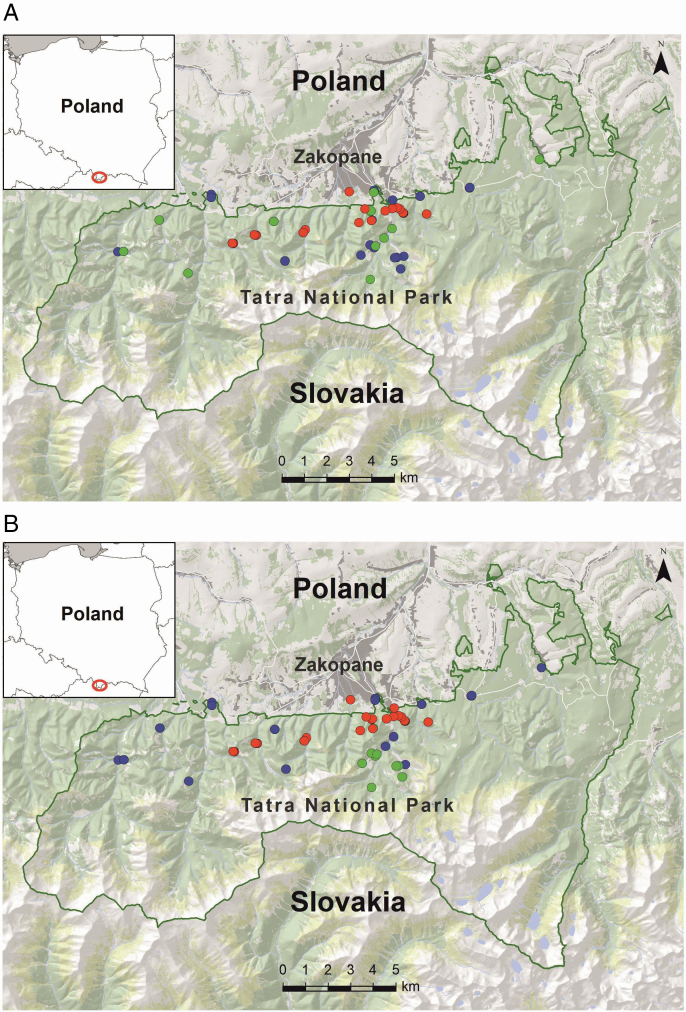
Geographical distribution of the studied trees of different *Sorbus* species from the Tatra National Park. The red circles indicate the location of *Sorbus aria* trees, blue—*S. intermedia* and green—*S. carpatica* assigned to the species before (A) and after genetic verification (B). Red empty circle indicates the location of the Tatra National Park on the map of Poland in the left upper corner of the figure.

In the summer of 2019, leaves from short shoots were collected from the middle part of the crown from 73 *Sorbus* trees from the TNP (for more details about TNP, please refer to Mirek 1996; Kempf *et al.* 2018; Zięba *et al.* 2018 and Supplementary Materials). Trees were chosen based on morphological classification, taking into account their accessibility in the mountain terrain, so that the collected samples come from populations of all *Sorbus* species reported in the TNP. Sampled trees were not visibly connected to each other to avoid sampling of clones. Fully expanded disease-free leaves were collected and stored dried until morphological analysis and DNA extraction. Based on the preliminary morphological identification performed after Szewczyk *et al.* (2011), the material of *S. aria* and *S. intermedia* trees was collected. Specimens exhibiting characteristics of other *Sorbus* species or initially identified as *S. intermedia*, but occurring far from a potential seed source and in close proximity to natural sites of *S. aria*, were preliminarily classified as *S. carpatica*. Information on the location of *Sorbus* individuals, sample size and methods used are presented in Table 1, and their geographical location is shown in Fig. 1.

**Table 1.** Geographical location of individuals collected in the Tatra National Park based on the morphological identification of the species (Species, Acronym) and correct assignment of individuals to species after genetic verification (Species 2, Acronym 2); n c—no changes; method used: MO—morphology analysis, SSR—genetic analysis. A—*S. aria*, J—*S. intermedia*, C—*S. carpatica* with following numbers of tested trees.

			Geographical coordinates			After genetic verification	
Species	Acronym	Geographical location	Longitude [E]	Latitude [N]	Method	Acronym 2	Species 2
*Sorbus intermedia*	J01	Biathlon	19.87	49.28	MO, SSR	n c	n c
	J02	Brzeziny	20.03	49.29	MO, SSR	n c	n c
	J03	Brzeziny	20.03	49.29	MO, SSR	n c	n c
	J04	Brzeziny	20.03	49.29	MO, SSR	n c	n c
	J05	Biathlon 2	19.87	49.28	MO, SSR	n c	n c
	J06	Biathlon 3	19.87	49.28	MO, SSR	n c	n c
	J07	Rondo 1	19.97	49.29	MO, SSR	n c	n c
	J08	Rondo 2	19.97	49.29	MO, SSR	n c	n c
	J09	Rondo 3	19.97	49.29	MO, SSR	n c	n c
	J10	Rondo 4	19.97	49.29	MO, SSR	n c	n c
	J11	Nosal 1	19.98	49.28	MO, SSR	A_J11	*S. aria*
	J12	Huciska	19.82	49.26	MO, SSR	n c	n c
	J13	Jaworzynka 1	19.99	49.26	MO, SSR	n c	n c
	J14	Jaworzynka 2	19.99	49.26	MO, SSR	C_J14	*S. carpatica*
	J15	Jaworzynka 3	19.98	49.26	MO, SSR	C_J15	*S. carpatica*
	J16	Jaworzynka 4	19.99	49.25	MO, SSR	C_J16	*S. carpatica*
	J17	Kalatówki 1	19.97	49.26	MO, SSR	C_J17	*S. carpatica*
	J18	Kalatówki 2	19.97	49.26	MO, SSR	C_J18	*S. carpatica*
	J19	Leontynówka 1	20.00	49.28	MO, SSR	n c	n c
	J20	Leontynówka 1	20.00	49.28	MO, SSR	n c	n c
	J21	Leontynówka 1	20.00	49.28	MO, SSR	n c	n c
	J22	Leontynówka 1	20.00	49.28	MO, SSR	n c	n c
	J23	Kalatówki 3	19.96	49.26	MO, SSR	C_J23	*S. carpatica*
	J24	Dolina nad Capkami 4	19.97	49.27	SSR	C_J24	*S. carpatica*
	J25	Kalatówki 4	19.97	49.26	MO, SSR	C_J25	*S. carpatica*
	J26	Grzybowiec 1	19.92	49.26	MO, SSR	n c	n c
	J27	Grzybowiec 2	19.92	49.26	MO, SSR	n c	n c
	J28	Grzybowiec 3	19.92	49.26	MO, SSR	n c	n c
	J29	Grzybowiec 4	19.92	49.26	MO, SSR	n c	n c
	J30	Grzybowiec 5	19.92	49.26	MO, SSR	n c	n c
*Sorbus carpatica*	C01	Błociska	20.07	49.30	MO, SSR	J_C01	*S. intermedia*
	C02	Kuźnice	19.98	49.27	MO, SSR	J_C02	*S. intermedia*
	C03	Mały Żlebek 1	19.91	49.27	MO, SSR	J_C03	*S. intermedia*
	C04	Mały Żlebek 1	19.91	49.27	MO, SSR	J_C04	*S. intermedia*
	C05	Mały Żlebek 1	19.91	49.27	MO, SSR	J_C05	*S. intermedia*
	C06	Mały Żlebek 1	19.91	49.27	MO, SSR	J_C06	*S. intermedia*
	C07	CEP	19.97	49.28	MO, SSR	J_C07	*S. intermedia*
	C08	Huciska pod Baniami	19.82	49.26	MO, SSR	J_C08	*S. intermedia*
	C09	Huciska pod Baniami	19.82	49.26	MO, SSR	J_C09	*S. intermedia*
	C10	Huciska pod Baniami	19.82	49.26	MO, SSR	J_C10	*S. intermedia*
	C11	Huciska pod Baniami	19.82	49.26	MO, SSR	J_C11	*S. intermedia*
	C12	Stoły	19.86	49.25	MO, SSR	J_C12	*S. intermedia*
	C13	Dolina nad Capkami 1	19.97	49.28	MO, SSR	A_C13	*S. aria*
	C14	Dolina nad Capkami 2	19.97	49.28	MO, SSR	A_C14	*S. aria*
	C15	Energetyk	19.98	49.27	MO, SSR	J_C15	*S. intermedia*
	C16	Goryczkowa	19.97	49.25	SSR	n c	n c
	C17	Kalatówki	19.97	49.26	MO, SSR	n c	n c
	C18	Cisowa Turnia	19.84	49.27	SSR	J_C18	*S. intermedia*
	C19	Cisowa Turnia	19.84	49.27	SSR	J_C19	*S. intermedia*
	C20	Dol. Strążyska	19.93	49.27	SSR	A_C20	*S. aria*
*Sorbus aria*	A01	Nosal 1	19.99	49.28	MO, SSR	n c	n c
	A02	Nosal 2	19.99	49.28	MO, SSR	n c	n c
	A03	Nosal 3	19.99	49.28	MO, SSR	n c	n c
	A04	Nosal 4	19.99	49.28	MO, SSR	n c	n c
	A05	Nosal 5	19.99	49.28	MO, SSR	n c	n c
	A06	Nosal 6	19.99	49.28	MO, SSR	n c	n c
	A07	Nosal 7	19.99	49.28	MO, SSR	n c	n c
	A08	Pod Nosalem	19.98	49.28	MO, SSR	n c	n c
	A09	Kogutki	19.97	49.28	MO, SSR	n c	n c
	A10	Dolina Białego 1	19.96	49.27	SSR	n c	n c
	A11	Kończysta T. 1	19.89	49.26	MO, SSR	n c	n c
	A12	Kończysta T. 2	19.89	49.26	MO, SSR	n c	n c
	A13	Kończysta T. 3	19.89	49.26	MO, SSR	n c	n c
	A14	Kończysta T. 4	19.89	49.26	MO, SSR	n c	n c
	A15	Dolina nad Capkami 3	19.97	49.27	MO, SSR	n c	n c
	A16	Dolina Białego 2	19.96	49.28	MO, SSR	n c	n c
	A17	Mały Kopieniec	20.00	49.28	MO, SSR	n c	n c
	A18	Gmińska Turnia 1	19.90	49.27	SSR	n c	n c
	A19	Gmińska Turnia 2	19.90	49.27	SSR	n c	n c
	A20	Gmińska Turnia 3	19.90	49.27	SSR	n c	n c
	A21	Krokiew	19.98	49.28	SSR	n c	n c
	A22	Dol. Strążyska 1	19.93	49.27	SSR	n c	n c
	A23	Dol. Strążyska 2	19.93	49.27	SSR	n c	n c

Genomic DNA was extracted from 200 mg of dried leaves using a commercial kit Genomic Midi AX Plant (A&A Biotechnology) according to the manufacturer’s instructions. The extraction efficiency was analysed by 1 % agarose gel electrophoresis and the quantity was checked using NanoDrop Life (Thermo Scientific). DNA extracts were diluted with nuclease-free water (A&A Biotechnology) to a concentration of 10–20 ng/µL for genetic analysis.

### Genetic analyses

Analysis of the genetic variability of *Sorbus* species was carried out using 13 nuclear microsatellite markers (*n*SSR—nuclear Simple Sequence Repeats) (Gianfranceschi *et al.* 1998; Oddou-Muratorio *et al.* 2001; Liebhard *et al.* 2002; Kamm *et al.* 2009; González-González *et al.* 2010). Primer sequences, PCR conditions and multiplex PCR protocols used in the analysis are listed in **Supporting Information—Table S1**. PCR products were separated on the ABI 3500 capillary sequencer (Applied Biosystems) and the length of each DNA fragment was sized relative to an internal size standard and calculated using GeneMapper software. Each allele peak designation was checked and confirmed manually. Any inconsistent samples were repeated to ensure the observed allele sizes were not artefacts or scoring errors.

### Genotypic diversity

Based on the allele distribution data at each locus and for each sample, different numbers of alleles were observed (two or three) depending on the locus and *Sorbus* species analysed. Because allele identification in polyploid individuals is complicated, comparison of multilocus genotypes was performed following Robertson *et al.* (2010). Ploidy level estimates for tested individuals were based on the maximum number of displayed alleles at a single locus. Therefore, the individual was considered as diploid when all 13 loci analysed showed the maximum two alleles. Consistently, the individual was considered as a polyploid when it contained three alleles at any of the loci sampled.

The tested loci were characterized by the number of alleles for all individuals (*N*_a_), Simpson index defined as 1 − *D* (Simpson 1949) and heterozygosity according to Nei (1978) (*H*_exp_). The following interspecies genetic parameters were calculated: MLG, number of multilocus genotypes, which was a unique combination of alleles in all the loci for the individual; eMLG, the number of expected MLG at the smallest sample size ≥ 10 based on rarefaction. The Stoddart and Taylor index (1988), marked as *G* was used to characterize the genetic variability describing the genetic structure within the species. This measure is less susceptible to different sample sizes and indicates what genotype frequency should be expected for each locus when analysing multiple loci simultaneously. It was calculated as a fraction of one by the sum of squares of frequencies of individual genotypes for multiple loci. The calculation of genetic parameters was carried out using the R 3.6.1 software (R Core Team 2019) and the poppr 2.8.3package (Kamvar *et al.* 2015; Grünwald *et al.* 2017).

### The standardized index of association, *ṝ*_d_

To check the ways of reproduction of the examined species of the *Sorbus* genus, an analysis of the association (*ṝ*_d_) was performed (Brown *et al.* 1980; Kamvar *et al.* 2014). This approach was used as *Sorbus* species may reproduce sexually through outbreeding, but also through apomixis (i.e. from unfertilized seeds) or clonally from root suckers. As the way of propagation may be related to the polyploidy of organisms, it was assumed that the imbalance of conjunctions occurs when alleles in two or more loci coexist more frequently than is predicted based on their frequency. The association index estimated based on the frequency of genotypes for randomly crossed populations is 0. Any statistically significant deviation from the expected zero would therefore suggest clonal reproduction. In this analysis, the significance was tested based on 999 permutations conducted in the R poppr package(Kamvar *et al.* 2014).

### Population structure

The genetic structure of the examined *Sorbus* species was analysed based on the allele frequency distribution in the tested loci. The genetic distance between pairs of all individuals was calculated following Bruvo *et al.* (2004). The neighbour-joining algorithm, based on Bruvo’s distance, was used to visualize the clustering of *Sorbus* species with 1000 bootstrap replicates. Then, the *K*-means clustering in combination with a bootstrapped dendrogram was used to demonstrate the patterns of genetic identity of the tested *Sorbus* species. *K*-means is a measure of group’s differentiation and relies on an equation which decomposes the total variance of a variable into between-group and within-group components (Liu and Zhao 2006; Lee *et al.* 2009; Jombart *et al.* 2010). We assumed that populations with clonal reproduction should have short terminal branch lengths and cluster together, while the sexually reproducing populations will show no clear pattern. The Minimum Spanning Network (MSN) was calculated based on the Bruvo distance and a stepwise mutation model (Kamvar *et al.* 2015). Additionally, to show the genetic relationships between *Sorbus* species, genetic distance based on all polymorphic genotypes was presented using Discriminant Analysis of Principal Components (DAPC) (Jombart *et al.* 2010; Grünwald and Goss 2011). In this analysis, data are first transformed using a principal component analysis (PCA) and subsequently clusters are identified using discriminant analysis (DA). This approach is more convenient than both Bayesian clustering and standard PCA for the analysis of populations that reproduce clonally or partially clonally (Jombart *et al.* 2010; Grünwald and Goss 2011). The analysis was carried out using the R 3.6.1 software (R Core Team 2019) and the poppr 2.8.3 package (Kamvar *et al.* 2015; Grünwald *et al.* 2017).

### Morphological analysis of leaves

**Figure 2.**
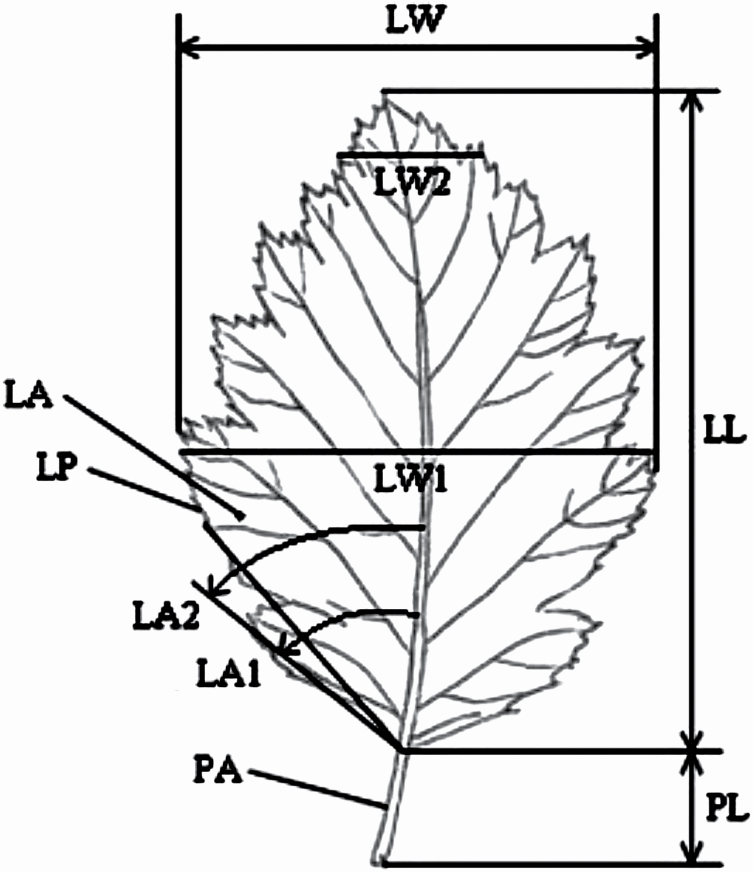
Measured leaf traits. LA—leaf area (cm^2^), LP—leaf perimeter (cm), LL—leaf length (cm), LW—maximum leaf width (cm), LW1—leaf blade width at 50 % of blade length, LW2—leaf blade width at 90 % of blade length (cm), LA1—angle closed by main vein and the line defined by the leaf blade base and a point on the margin, at 10 % of blade length, LA2—angle closed by main vein and the line defined by the leaf blade base and a point on the margin, at 25 % of blade length, PL—petiole length, (cm), PA—petiole area, (cm^2^) and FC—form coefficient—numerical value which grades the leaf shape between circular (shortest perimeter for a given area) and filiform (longest perimeter for a given area) and LW/LL and PL/LL.

Ten metric features and three parameters calculated from the obtained measurements were used for the morphometric analysis (Fig. 2). The parameters were estimated with an accuracy of 1 μm using WinFOLIA Reg 2018 software (Regent Instruments Inc., Quebec, Canada). Arithmetic means (*M*), minimum and maximum values (Min., Max.), standard deviation (SD) and coefficient of variation (CV) were calculated for the morphometric features. The significance was checked using non-parametric tests. The results of Shapiro–Wilk and Levene’s test led to the use of ANOVA test of Kruskal–Wallis rank. To assess the grouping factors, the analysis of principal components (PCA) for standardized data was applied. The statistical analysis was performed using Excel (2013) and Statistica 13.3 software.

## Results

The 13 microsatellite loci provided a set of 110 unique alleles within the tested *Sorbus* population. The number of observed alleles at different loci ranged from 3 to 15, with an average of 8.5 (Table 2). Analysed *Sorbus* species showed a different number of alleles for the studied loci **[see**  **Supporting Information—Fig. S1****]**. Loci SA07, CH01h10, SA02, MS14h03, SA09 and SA19.1 had two alleles in all tested individuals. The remaining SA01, SA06, MSS5, SA08, CH01h01, CH02C09 and SA14 loci showed two alleles in all individuals of *S. aria* and two or three alleles in individuals of the other species. Locus CH01h10 failed to amplify in *S. aria* individuals. Overall, the microsatellite loci show different patterns of allele distribution in the studied species and can therefore be used for the identification of *Sorbus* taxa.

**Table 2.** Genetic parameters for the nuclear microsatellite loci tested in *Sorbus* species. *N*_a_—number of alleles for all tested individuals; 1 − *D*—Simpson index (Simpson 1949); *H*_exp_—heterozygosity (Nei 1978).

Locus	*N*_a_	1 − *D*	*H*_exp_
MSS5	8	0.772	0.778
SA01	11	0.829	0.834
SA06	15	0.869	0.874
SA07	5	0.534	0.538
CH01h01	9	0.774	0.778
CH01h10	3	0.406	0.410
SA02	9	0.784	0.789
SA08	11	0.836	0.841
CH02c09	7	0.769	0.773
MS14H03	3	0.056	0.056
SA09	9	0.764	0.769
SA14	13	0.845	0.850
SA19	7	0.733	0.739
Mean	8.46	0.690	0.695

### Verification and genetic annotation for the analysed *Sorbus* specimens

**Figure 3.**
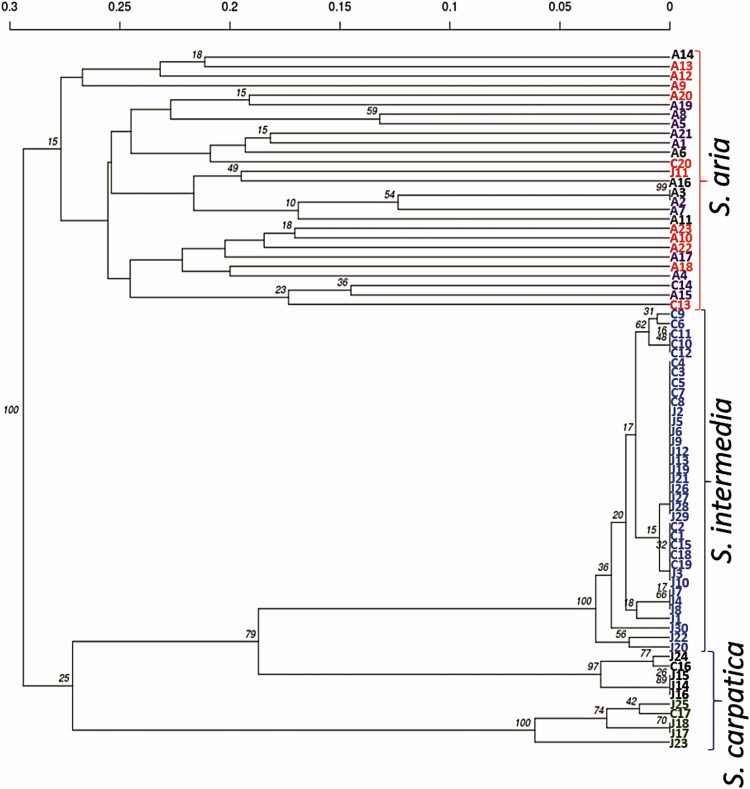
Dendrogram of genetic distances (Bruvo *et al.* 2004) based on nuclear microsatellite loci in *Sorbus* genus. A1–23, C1–20, J1–30—individual acronym of species (A—*S. aria*, C—*S. carpatica*, J—*S. intermedia*). Large rectangle—clusters for *K* = 3, different colours of individuals acronyms show the results of cluster analysis for *K* = 6.

Different distribution of alleles in the studied loci and species indicated that some individuals were assigned to a wrong taxon based on morphological identification in the field. Based on genetic distance and the dendrogram, it was possible to identify groups corresponding to particular *Sorbus* species. The tested individuals were assigned to three main groups (clusters), corresponding to the examined species: *S. aria*, *S. carpatica* and *S. intermedia* (Fig. 3). A detailed analysis of the assignment of individuals to particular clusters indicated intrataxon genetic heterogeneity (Fig. 3). The group of individuals representing *S. intermedia* included individuals J1–10, J12–13, J19–22, J26–30 and C1–12, C15, C18–19, which were originally assigned to *S. carpatica* (see Fig. 3, trees marked in blue). For *K* = 3, the trees grouped genetically to *S. aria* included all originally selected individuals of this species (A1–23), and additionally trees C13, C14 and C20, representing originally *S. carpatica* and J11 initially assigned as *S. intermedia* (Fig. 3, *S. aria* individuals are marked with a red rectangle). When analysing a larger number of clusters (*K* = 6), the *S. aria* group was divided into three smaller groups. This division may have originated from the large genetic distance of *S. aria* individuals, high variability and non-panmictic reproduction (Fig. 3, trees marked in violet, red and grey). Based on the dendrogram, the *S. carpatica* group was formed by trees: C16–17 originally assigned to this group and J14–18 and J23–25 originally assigned to *S. intermedia*. For *K* = 6, this group turned out to be heterogeneous and was divided into two: one with trees J17–18, J23, J25, C17 and the other with trees J14–16, J24, C16 (Fig. 3, trees marked in green and black, respectively). This suggests that genetic analysis enables discrimination of *S. intermedia* and *S. carpatica.*

**Figure 4.**
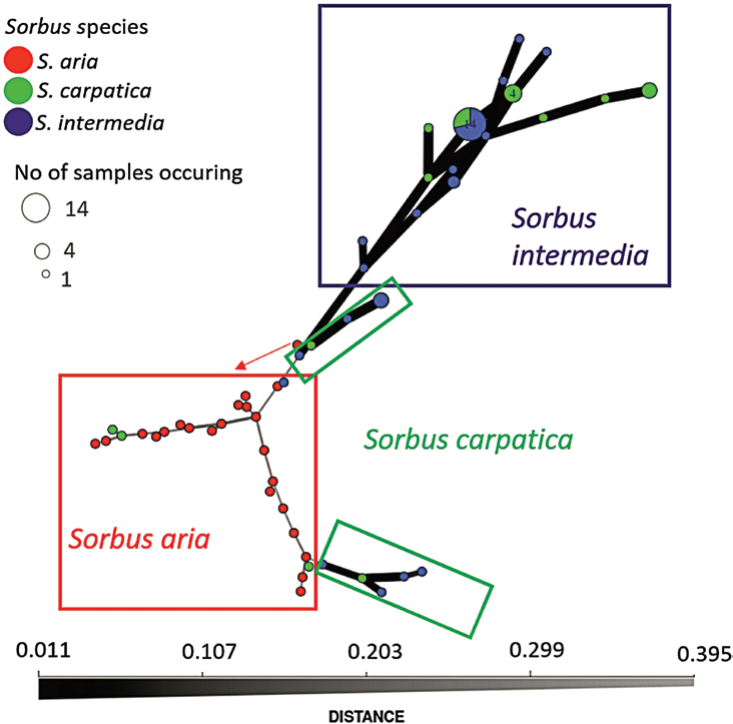
Minimum spanning network (MSN) based on Bruvo’s genetic distance for nuclear microsatellite loci for *Sorbus* genus. Circles represent individual MLG (number of multilocus genotypes), colours represent taxa membership: blue individuals of *S. intermedia*, red—*S. aria*, green—*S. carpatica*. Circle sizes are proportional to the number of samples: the largest circles include 14 individuals, small—four and the smallest circle includes one individual. Lines represent the minimum genetic distance between individuals. Circles that are more closely related have darker and thicker edges, whereas circles more distantly related have lighter and thinner edges (Kamvar *et al.* 2015).

To validate the initial genetic assignment of *Sorbus* individuals (Fig. 3), we performed a MSN analysis (Fig. 4). The network of genetic relationships presented a group of *S. aria* individuals (red circles) spatially distributed on four narrow branches indicating their greatest genetic distance. On the same branches, we found some of the *S. carpatica* individuals (green circles) and *S. intermedia* (blue circles) wrongly annotated in morphological analysis. Individuals of *S. intermedia* constituted a single group with connections with other individuals, initially erroneously assigned morphologically as representatives of *S. carpatica*. In MSN analysis, the hybrids were divided into two groups, which were in line with the results of the dendrogram for *K* = 6.

### Variability and genetic structure of *S. aria, S. intermedia* and their hybrids

Genetic analyses assigned individual specimens to *S. aria* (27 trees: A1–23, C13–14, C20, J11), *S. intermedia* (36 trees: J1–10, J12–13, J19–22, J26–30, C1–12, C15, C18–19) and *S. carpatica* hybrids (10 trees: C16–17, J15–18 and J23–25). For *S. aria*, 27 different genotypes were identified for the 27 examined trees. For *S. carpatica* and *S. intermedia* the number of genotypes was lower (Table 3). When the number of examined individuals per species (eMGL) was taken into account, the number of expected genotypes was the lowest for *S. intermedia*. *Sorbus aria* was characterized by the highest genetic variability, visualized by the *G* parameter equal to 27. In contrast, *S. intermedia* and *S. carpatica* exhibited similar, lower level of genetic variability with *G* equal to 5.45 and to 6.25, respectively. Heterozygosity was highest for *S. aria* (*H*_exp_ = 0.623) and lowest for *S. intermedia* (*H*_exp_ = 0.481). Based on the association index (*ṝ*_d_ = 0.0689), interbreeding was found to be the main reproduction strategy for *S. aria*. In contrast, *S. carpatica* had an association index significantly different from zero, suggesting that it mainly utilizes clonal reproduction (Table 3). Unfortunately, given the small number of alleles for the analysed loci, no reliable estimates of the association index could be obtained for *S. intermedia.* Overall, different patterns of genetic variation and mating systems were observed for the analysed *Sorbus* species.

**Table 3.** The parameters of genetic variability calculated for the tested *Sorbus* genus. Description of abbreviations as in the text. *N*_a_—number of alleles for all tested individuals; MLG—number of multilocus genotypes; eMLG—number of expected MLG at the smallest sample size ≥ 10 based on rarefaction; SE—standard error for eMLG; *G*—Stoddart and Taylor index (1988); *H*_exp_—heterozygosity (Nei 1978); *ṝ*_d_—standardized index of association; *P-*value for *ṝ*_d_—statistical significance.

Species	*N*_a_	MLG	eMLG	SE	*G*	*H*_exp_	*ṝ*_d_	*P-*value for *ṝ*_d_
*S. aria*	27	27	10.00	0.00	27.00	0.623	0.069	*P* = 0.06
*S. intermedia*	36	17	6.47	1.27	5.45	0.481	−0.014	*P* = 1
*S. carpatica*	10	8	8.00	0.00	6.25	0.612	0.824	*P* = 0.01
Sum/mean	73	52	8.77		18.96	0.695	0.470	

To evaluate the genetic structure of the *Sorbus* species tested, DAPC analysis was carried out **[see**  **Supporting Information—Fig. S2****]**. It showed that the tested individuals could be assigned to three clearly differentiated species. *Sorbus aria* created the most diverse group which was also separated by the greatest distance from the other species. Results of the DAPC analysis supported the genetic structure showed on dendrogram (Fig. 3) or in MSN analysis (Fig. 4).

### Variability of leaf morphology

Leaf morphology showed some differences between the specimens (Table 4), but no specific traits allowed for their reliable taxonomic identification. Individuals of *S. aria* had leaves with the highest size parameters (LA, LL, LW), with the smallest average circumference (LP), which is the effect of shallow serration of leaf blades of this species. The leaves of *S. intermedia* had the longest and thickest petioles (PL, PA) and the deepest leaf blade serration (LP). The leaves of *S. carpatica* were characterized by the smallest parameters of leaf (LA, LL, LW) and petiole size (PL, PA). The highest intraspecies variability (above 20 %) was observed for such parameters as: LA, LP, LW2, PL and PA, especially for *S. aria* and *S. intermedia*. Leaf area (LA), maximum leaf blade width (LW) and leaf blade width at 50 % of the length (LW1) differentiated most *S. carpatica* from *S. aria* and *S. intermedia* based on Kruskal–Wallis test following ANOVA (Table 5). The circumference of the leaf blade (LP), the angle between the main nerve and the point on the edge of the blade at 25 % of the leaf length (LA2), the length of the petiole (PL) and the width-to-length ratio of the blade (LW/LL) were the features that most differentiated *S. intermedia* from the other two species. *Sorbus aria* differed from *S. carpatica* and *S. intermedia* by the angle between the main nerve and a point on the edge of the leaf blade located at 10 % of the leaf length (LA1) and the width of the leaf blade at 90 % of its length (LW2). However, biometric analysis of leaves did not allow us to identify diagnostic markers for faultless delimitation of the *Sorbus* species.

**Table 4.** Descriptive statistic of the measured morphological traits of *Sorbus* genus. *N*—number of individuals, LA—leaf area, LP—leaf perimeter, LL—leaf length, FC—form coefficient, LW—maximum leaf width, LW1—leaf blade width at 50 % of blade length, LW2—leaf blade width at 90 % of blade length, LA1—angle closed by main vein and the line defined by the leaf blade base and a point on the margin, at 10 % of blade length, LA2—angle closed by main vein and the line defined by the leaf blade base and a point on the margin, at 25 % of blade length, PL—petiole length, PA—petiole area, LW/LL and PL/LL, statistical measures in bold/italic: *M*—arithmetic means, Min., Max.—minimum and maximum values, SD—standard deviation, CV—coefficient of variation. A—*S. aria*, J—*S. intermedia*, C—*S. carpatica* with following numbers of tested trees.

No	Species	*N*	LA (cm^2^)	LP (cm)	FC	LL (cm)	LW (cm)	LW1 (cm)	LW2 (cm)	LA1 (°)	LA2 (°)	PL (cm)	PA (cm^2^)	LW/LL	PL/LL
1	A01	25	26.88	25.32	0.58	8.01	4.63	4.51	2.44	44.36	39.08	1.41	0.17	0.58	0.18
2	A02	20	30.85	26.49	0.53	7.98	5.21	5.08	2.48	56.50	44.90	1.90	0.25	0.65	0.24
3	A03	29	25.42	23.47	0.55	6.95	4.76	4.65	2.28	58.14	46.79	1.45	0.17	0.68	0.21
4	A04	25	15.75	23.38	0.35	6.00	3.61	3.48	1.26	52.40	42.04	1.25	0.14	0.60	0.21
5	A05	20	45.24	39.30	0.33	9.68	6.35	6.11	2.35	51.50	44.55	1.29	0.15	0.66	0.13
6	A06	20	29.85	30.92	0.36	7.99	5.07	4.83	2.05	50.90	43.60	1.40	0.18	0.63	0.18
7	A07	19	45.49	34.81	0.47	10.00	6.50	6.27	3.23	47.53	42.84	2.02	0.25	0.65	0.20
8	A08	31	42.44	34.71	0.40	9.31	6.05	5.88	2.61	52.06	44.42	1.44	0.15	0.65	0.15
9	A09	11	23.95	26.56	0.41	6.89	4.69	4.47	2.07	56.55	46.64	1.16	0.11	0.68	0.17
10	A11	21	25.11	31.40	0.31	7.41	4.72	4.53	1.63	56.57	46.00	1.28	0.14	0.64	0.17
11	A12	25	36.00	31.97	0.45	8.32	5.95	5.81	2.48	60.72	49.28	1.91	0.23	0.72	0.23
12	A13	13	41.89	36.50	0.38	10.45	5.84	5.70	2.34	44.62	38.00	2.10	0.23	0.56	0.20
13	A14	14	60.19	44.68	0.37	11.35	7.67	7.40	2.65	54.57	46.00	1.57	0.21	0.68	0.14
14	A15	10	30.64	26.94	0.47	8.26	4.75	4.55	1.98	50.50	40.50	1.51	0.18	0.58	0.18
15	A16	9	39.98	30.08	0.54	9.49	5.88	5.81	2.85	48.00	42.00	1.77	0.23	0.62	0.19
16	A17	15	24.84	25.66	0.46	7.98	4.42	4.27	1.78	47.27	40.07	0.87	0.08	0.55	0.11
17	A18	11	21.20	24.19	0.46	6.54	4.17	3.97	1.35	54.00	44.27	1.26	0.11	0.64	0.19
18	A19	11	51.84	44.47	0.32	11.80	6.38	6.06	2.49	46.09	38.55	1.65	0.16	0.54	0.14
19	A20	22	30.16	25.73	0.52	7.95	4.89	4.72	2.03	52.36	43.95	1.38	0.14	0.62	0.17
***M***			***34.09***	***30.87***	***0.44***	***8.55***	***5.34***	***5.16***	***2.23***	***51.82***	***43.34***	***1.51***	***0.17***	***0.63***	***0.18***
***Min.***			***15.75***	***23.38***	***0.31***	***6.00***	***3.61***	***3.48***	***1.26***	***44.36***	***38.00***	***0.87***	***0.08***	***0.54***	***0.11***
***Max.***			***60.19***	***44.68***	***0.58***	***11.80***	***7.67***	***7.40***	***3.23***	***60.72***	***49.28***	***2.10***	***0.25***	***0.72***	***0.24***
***SD***			***11.18***	***6.50***	***0.08***	***1.55***	***0.97***	***0.94***	***0.48***	***4.56***	***3.00***	***0.31***	***0.05***	***0.05***	***0.03***
***CV***			***32.79***	***21.07***	***18.92***	***18.17***	***18.12***	***18.27***	***21.60***	***8.81***	***6.92***	***20.58***	***27.53***	***7.57***	***18.16***
1	J01	31	36.54	43.96	0.24	8.57	6.25	5.93	2.13	59.58	49.55	2.28	0.27	0.73	0.27
2	J02	28	26.35	34.37	0.28	7.36	5.26	5.01	1.96	54.50	47.89	1.75	0.19	0.71	0.24
3	J03	30	27.36	42.27	0.19	7.51	5.38	4.93	1.57	60.40	48.73	1.55	0.16	0.72	0.21
4	J04	29	30.17	43.66	0.20	8.33	5.52	5.15	1.68	56.79	46.45	1.93	0.20	0.66	0.23
5	J05	22	30.97	43.78	0.20	8.06	5.57	5.20	2.00	55.55	46.36	1.76	0.19	0.69	0.22
6	J06	30	32.75	41.73	0.23	8.22	5.90	5.54	1.97	55.97	48.33	2.27	0.28	0.72	0.28
7	J07	18	40.15	46.62	0.23	9.24	6.48	6.08	2.03	58.28	49.11	2.20	0.27	0.70	0.24
8	J08	20	35.45	42.36	0.24	8.33	6.14	5.84	2.25	55.80	49.10	2.01	0.23	0.74	0.24
9	J09	30	39.32	46.54	0.22	8.73	6.76	6.25	2.27	57.73	51.50	1.87	0.21	0.77	0.21
10	J10	21	30.70	39.81	0.24	7.89	5.82	5.43	1.89	55.81	50.29	1.69	0.17	0.74	0.21
11	J12	32	22.31	32.75	0.26	7.06	4.60	4.30	1.50	53.44	45.03	2.11	0.25	0.65	0.30
12	J13	5	16.63	38.91	0.14	6.32	4.15	3.79	1.11	59.80	46.00	1.31	0.10	0.66	0.21
13	J19	31	25.83	36.81	0.24	7.17	5.33	4.99	1.85	57.68	49.48	1.79	0.20	0.74	0.25
14	J20	26	33.07	41.11	0.27	8.31	5.83	5.42	1.90	58.08	48.27	2.29	0.27	0.70	0.28
15	J21	18	38.93	44.73	0.25	9.04	6.47	6.06	2.09	55.06	47.39	2.14	0.24	0.72	0.24
16	J22	36	24.06	33.60	0.27	7.08	4.93	4.60	1.61	56.56	47.25	2.10	0.24	0.70	0.30
17	J26	20	22.97	34.26	0.24	6.98	4.86	4.68	1.54	57.75	47.40	2.16	0.25	0.70	0.31
18	J27	21	30.66	42.61	0.21	8.23	5.53	5.12	1.79	57.19	46.33	1.98	0.23	0.67	0.24
19	J28	20	35.56	43.60	0.23	8.77	5.96	5.65	2.04	57.65	46.55	2.57	0.32	0.68	0.29
20	J29	23	28.31	38.48	0.24	7.83	5.29	5.02	1.81	56.57	46.74	1.99	0.23	0.68	0.25
21	J30	20	42.08	51.29	0.20	9.47	6.59	6.26	2.19	57.80	48.15	2.20	0.26	0.70	0.23
22	J31	41	26.38	37.44	0.25	7.70	5.03	4.65	1.75	56.88	45.78	1.99	0.21	0.65	0.26
23	J32	22	32.26	40.57	0.23	8.32	5.50	5.07	1.84	55.09	45.09	2.01	0.24	0.66	0.24
24	J33	18	17.56	29.27	0.24	6.07	4.14	3.79	1.36	60.72	46.39	1.42	0.12	0.68	0.23
25	J34	8	11.50	23.25	0.25	5.12	3.17	2.98	1.00	55.50	44.88	0.95	0.07	0.62	0.19
26	J35	4	15.35	27.83	0.25	5.70	4.00	3.46	1.40	63.25	46.75	1.19	0.10	0.70	0.21
27	J36	6	19.66	33.38	0.22	6.41	4.46	4.23	1.24	63.00	48.17	1.30	0.11	0.70	0.20
28	J37	30	42.82	46.03	0.24	9.32	6.54	6.06	2.15	56.53	47.80	2.39	0.31	0.70	0.26
29	J38	27	24.34	41.79	0.17	7.11	4.93	4.45	1.58	59.78	46.52	1.55	0.13	0.69	0.22
30	J39	2	5.33	17.62	0.21	3.97	2.15	1.92	0.40	50.00	43.50	0.97	0.05	0.54	0.24
31	J40	2	7.85	24.75	0.16	4.17	3.02	2.64	0.83	64.00	49.00	1.75	0.12	0.72	0.42
32	J41	3	14.84	36.37	0.14	6.09	3.95	3.67	0.96	60.00	46.33	1.11	0.08	0.65	0.18
33	J42	42	17.58	30.03	0.24	5.84	4.21	3.94	1.35	63.02	49.02	1.81	0.19	0.72	0.31
34	J43	37	35.45	51.99	0.16	8.93	6.23	5.52	1.45	61.54	47.41	2.50	0.29	0.70	0.28
***M***			***27.09***	***38.34***	***0.22***	***7.45***	***5.17***	***4.81***	***1.66***	***57.86***	***47.43***	***1.85***	***0.20***	***0.69***	***0.25***
***Min.***			***5.33***	***17.62***	***0.14***	***3.97***	***2.15***	***1.92***	***0.40***	***50.00***	***43.50***	***0.95***	***0.05***	***0.54***	***0.18***
***Max.***			***42.82***	***51.99***	***0.28***	***9.47***	***6.76***	***6.26***	***2.27***	***64.00***	***51.50***	***2.57***	***0.32***	***0.77***	***0.42***
***SD***			***9.64***	***7.70***	***0.04***	***1.39***	***1.09***	***1.05***	***0.44***	***2.98***	***1.67***	***0.42***	***0.07***	***0.04***	***0.04***
***CV***			***35.58***	***20.09***	***15.85***	***18.69***	***21.08***	***21.81***	***26.34***	***5.15***	***3.52***	***22.78***	***35.81***	***5.90***	***17.83***
1	C1	19	18.04	25.94	0.31	6.08	3.96	3.70	1.50	59.11	43.63	1.41	0.11	0.65	0.23
2	C2	15	19.58	29.74	0.27	6.81	4.03	3.79	1.49	55.20	41.00	1.18	0.11	0.59	0.17
3	C3	22	23.26	33.76	0.24	7.35	4.44	4.08	1.60	54.45	40.91	1.46	0.14	0.60	0.20
4	C4	30	22.52	35.55	0.22	6.97	4.57	4.06	1.71	60.73	42.37	1.55	0.16	0.66	0.22
5	C5	26	19.14	29.95	0.28	6.42	4.41	3.91	1.54	62.65	45.65	1.50	0.16	0.69	0.23
6	C6	9	20.25	30.72	0.26	6.68	4.18	3.86	1.76	59.78	43.33	1.40	0.13	0.63	0.21
7	C7	16	30.64	39.91	0.24	8.64	5.44	4.97	1.93	56.13	40.69	1.84	0.16	0.63	0.21
8	C8	15	26.78	34.78	0.29	7.59	5.12	4.81	2.04	62.07	44.80	1.31	0.11	0.68	0.17
***M***			***22.53***	***32.54***	***0.26***	***7.07***	***4.52***	***4.15***	***1.70***	***58.76***	***42.80***	***1.46***	***0.14***	***0.64***	***0.21***
***Min.***			***18.04***	***25.94***	***0.22***	***6.08***	***3.96***	***3.70***	***1.49***	***54.45***	***40.69***	***1.18***	***0.11***	***0.59***	***0.17***
***Max.***			***30.64***	***39.91***	***0.31***	***8.64***	***5.44***	***4.97***	***2.04***	***62.65***	***45.65***	***1.84***	***0.16***	***0.69***	***0.23***
***SD***			***4.03***	***4.05***	***0.03***	***0.75***	***0.49***	***0.45***	***0.19***	***2.94***	***1.75***	***0.18***	***0.02***	***0.03***	***0.02***
***CV***			***17.89***	***12.45***	***10.27***	***10.54***	***10.82***	***10.79***	***11.21***	***5.01***	***4.10***	***12.45***	***17.01***	***4.87***	***10.75***

**Table 5.** Values of the Kruskal–Wallis test for measured morphological traits. LA—leaf area (cm^2^), LP—leaf perimeter (cm), FC—form coefficient, LL—leaf length (cm), LW—maximum leaf width (cm), LW1—leaf blade width at 50 % of blade length, LW2—leaf blade width at 90 % of blade length (cm), LA1—angle closed by main vein and the line defined by the leaf blade base and a point on the margin, at 10 % of blade length, LA2—angle closed by main vein and the line defined by the leaf blade base and a point on the margin, at 25 % of blade length, PL—petiole length (cm), PA—petiole area (cm^2^). *H* values for analysed traits; c2 test statistics. Results of multiple comparisons are presented in last tree columns, which includes the corresponding *z*-values for tested species (A—*S. aria*, J—*S. intermedia*, C—*S. carpatica*) for which significant levels are showed: ^ns^*P* > 0.05, ***P ≤ 0.0014.

Trait	*H*	c2	A–J	A–C	J–C
LA	41.86	19.43	2.02ns	6.39***	5.51***
LP	188.41	129.10	13.08***	2.26ns	7.04***
FC	679.13	457.86	26.04***	12.72***	5.03***
LL	44.02	20.46	3.64***	6.60***	4.56***
LW	44.92	20.58	1.99ns	4.80***	6.69***
LW1	55.14	34.79	0.30ns	6.47***	7.29***
LW2	75.01	42.43	7.65***	7.09***	2.17ns
LA1	113.94	78.45	9.91***	8.01***	1.54ns
LA2	159.49	123.05	10.17***	2.02ns	9.60***
PL	209.11	159.21	12.89***	0.03ns	9.34***
PA	148.47	113.79	7.89***	4.69***	10.85***
LW/LL	135.70	101.65	10.73***	0.85ns	6.88***
PL/LL	346.15	267.53	18.34***	5.57***	7.25***

The PCA was used to establish if leaf features can be used to assign the taxa. Principal component analysis showed the existence of four factors meeting the Kaiser criterion, with own values greater than 1, which together explained 93.52 % of the variance. The first component, which explained 52.85 % of the variance, and the second one, which explained 19.72 % of the variance **[see**  **Supporting Information—Table S3****]**, had the greatest contribution to explaining the observed variability **[see**  **Supporting Information—Fig. S3****]**. The first component showed a negative correlation with all analysed features, except for the angle between the main nerve and a point on the edge of the leaf blade located at 10 % of the leaf length (LA1). This means that the samples located in the figure on the right side of the *x*-axis were characterized by low average values of almost all analysed leaf parameters. The first principal component was influenced by the maximum width of the leaf blade (LW), the width of the leaf blade measured at 50 % of the leaf blade length (LW1), the area (LA) and length of the leaf blade (LL) and the width of the leaf blade measured at 90 % of the leaf blade length (LW2). The second component was influenced by features related to the angle between the main nerve and the point on the edge of the leaf blade located at 10 % and 90 % of the leaf length (LA1 and LA2) and the width-to-length ratio of the leaf blade (LW/LL). The PCA did not show the grouping of individuals from a particular species in relation to the analysed factors.

## Discussion

### Genetic and morphological features

We used genetic and morphological data to assess the diversity of the *Sorbus* genus within the TNP. In our analysis, we have studied the native, hybrid and foreign *Sorbus* species. The genetic analysis allowed for discrimination of *S. aria* and *S. intermedia* individuals and indicated the existence of their potential hybrid form *S. carpatica*. Patterns of variability at nuclear microsatellite loci confirmed that *S. aria* in the TNP is diploid. The observed population genetics parameters for *S. aria* in the Tatra Mountains are similar to these of individuals from the Iberian Peninsula, where the selected loci also had two alleles (Sosa *et al.* 2014). Moreover, the studied *S. aria* population has high genetic variability and high level of differentiation. Importantly, our analysis indicated that each *S. aria* individual had a unique genotype (MGL). This high genetic variability, together with the cross-propagation system, confirmed by our study, is characteristic mainly for diploid *Sorbus* species (Ludwig *et al.* 2013; Hamston *et al.* 2018). Although both diploid and triploid populations of *S. aria* have been found in Central Europe (Ludwig *et al.* 2013; Feulner *et al.* 2017), the observed pattern of genetic variability and structure of the tested population of *S. aria* is a consequence of interbreeding of diploid genomes.

The other species, including *S. intermedia*, artificially introduced into TNP, and *S. carpatica*, an endemic hybrid form, exhibited typical features of polyploid organisms. They had limited genetic variability, reproduced mainly through clonal propagation and had two to three alleles at the loci tested. *Sorbus intermedia* is considered a tetraploid species (Nelson-Jones *et al.* 2002; Robertson *et al.* 2010), but in most of the loci it shows three alleles (Robertson *et al.* 2010; this study). The number of discrete alleles detected in a polyploid individual, like a tetraploid *S. intermedia*, is often lower than its ploidy level (Robertson *et al.* 2010; Sosa *et al.* 2014). Thus, the studying of genetic diversity for polyploid and apomictic taxa relies on a comparison of multilocus genetic phenotypes (Robertson *et al.* 2010). Homogeneous structure of *S. intermedia* and the occurrence of dominant genotypes may result from strong selection pressure, favouring only genotypes adapted to the difficult environment of the Polish Tatra Mountains. The clonal system of reproduction stabilizes these genotypes and the occasional cross-fertilization (Robertson *et al.* 2010; Ludwig *et al.* 2013) enables interspecies hybridization and formation of heterotic progeny.

*Sorbus carpatica* is likely an endemic form, historically recognized by its leaf morphology, being intermediate between that of *S. aria* and *S. intermedia*. In our study, *S. carpatica* was identified as individuals with *S. intermedia* leaf features, but located far from the source of the *S. intermedia* seeds and close to the natural population of *S. aria*. Our genetic analyses of *n*SSR markers allowed correct assignment of individual trees to *Sorbus* species and identification of hybrid forms. The results confirmed the presence of both *S. aria* and *S. intermedia* species-specific alleles in *S. carpatica* genome. The investigated hybrid form probably has a triploid genome, which would result from the mating of diploid *S. aria* and tetraploid *S. intermedia*. *Sorbus carpatica* has a lower genetic variability than *S. aria* but greater than *S. intermedia*. Our results indicated that *S. carpatica* reproduces clonally, what is typical for triploid species (Ludwig *et al.* 2013).

The analysis of leaf size and shape showed that it is very difficult to distinguish the species solely based on morphological characteristics. In line with previous studies, we struggled to differentiate *S. intermedia* away from *S. carpatica* (e.g. Anamthawat-Jónsson and Thórsson 2003; Hynynen *et al.* 2010). Using selected morphological features (leaf area, leaf length, maximum leaf width), *S. aria* can be successfully differentiated from the other two species. During the collection of research material, 18 trees were annotated as *S. carpatica* but 80 % of them were confirmed to be actually *S. intermedia* after genetic analysis. These results, together with other literature reports (Minder *et al.* 2007), show that in case of hybrids of related species morphological analysis is not sufficient for correct species verification. The results are of particular importance for protected areas, such as the TNP, where identification of hybrids based on morphological features is a common practice during conservation-related field work.

### Mating systems

Native *S. aria* and foreign *S. intermedia* are both present in the TNP. Their common occurrence facilitates species hybridization. In case of diploid *S. aria* propagating through cross-fertilization, we can expect that the species is a donor of pollen in the process of hybridization. On the other hand, a tetraploid and clonally propagating *S. intermedia* is likely the dominant species in the hybridization process. According to Ludwig and colleagues (2013), tetraploid apomictic taxa can use their own pollen to create endosperms, so that the process of hybridization depends on the extent to which apomixis is optional. A very low level of genetic variability in the examined *S. intermedia* individuals may indicate a frequent occurrence of self-fertilization. However, the presence of hybrids involving *S. intermedia*, such as *S. carpatica*, and others (Liljefors 1954, 1955), indicates that this species can reproduce sexually through cross-fertilization under certain environmental conditions. This process increases the potential for interspecific hybridization. As such, it can be both beneficial and detrimental to ecosystem management, depending on the objectives pursed. One strategy is to protect the environment by maximizing the genetic diversity and potential for further hybridization (Ennos *et al.* 2012). It is believed this may increase the adaptability of the ecosystem to changing conditions. Another strategy, more common for officially protected areas and applicable in the TNP, regulated by Polish Regulation of Ministry of Environment of 28 December 2018 and Regulation of Ministry of Climate of 13 January 2020, is to maintain the genetic purity of natural populations. In this case, actions should be directed at eliminating the potential for hybridization between foreign species and native taxa.

Interestingly, in our analysis, *S. carpatica* hybrids were divided into two clusters. The first one showed features indicative of natural hybridization of *S. aria* and *S. intermedia* (green, Fig. 3). The second cluster preserved the bond with *S. intermedia*. These hybrids grow with a regular spacing, what most likely suggests that they were planted (black, Fig. 3). In that case, the seeds used to grow the propagation material were contaminated with hybrids already at the stage of harvesting. Planted seeds were most likely collected from trees growing in urbanized areas (Stecki 1952; Mirek 2016) what suggests that the hybridization process is not limited to wild individuals, but also may include trees from nearby urban greenery.

### Evolutionary implications

High genetic variability and absence of some loci, e.g. locus CH01h10, in *S. aria* in the Tatra Mountains suggests a long population history and its possible survival in that area during the last glaciation event as suggested for other forest tree species (Rull 2010; Stewart *et al.* 2010). Evolutionary success of *S. aria* may be associated with a unique genetic variability of its individuals, which in turn affects the potential to produce progeny with higher fitness. In addition, *S. aria* is involved in interspecific hybridization, which can lead to rapid genomic changes. These changes can result in favourable phenotypes, and selection for fertility and ecological traits can in turn change the structure of the genome (Baack and Rieseberg 2007). The combination of genetic variability, sexual reproduction and good performance of the diploid *S. aria* suggests that the species has a potential to adapt to the changing environment of TNP.

*Sorbus intermedia* is foreign to Tatra Mountains which was planted at the end of the 20th century as a biocenotic admixture in freshly forested areas (Stecki 1952; Mirek 2016). *Sorbus intermedia* individuals can be found along roadsides and near buildings at the lower elevation of the TNP, where they act as a source of seeds for ornitochory leading to occurrence of the species in different, often isolated, parts of the national park (Pusz *et al.* 2019). Due to the artificial origin of *S. intermedia* and lack of horticultural application, the species is not protected. Analysis of the health status of selected *S. intermedia* sites in TNP showed leaves colonized by, e.g., *Alternalia alternata* and *Boeremia exigua*, which are secondary pathogens occurring in tissues injured by others biotic or abiotic factors (Pusz *et al.* 2019). The observations suggest that *S. intermedia* has not adapted to the environment conditions of TPN, despite the fact that this species is considered to tolerate unfavourable habitats (Sjöman *et al.* 2016). Susceptibility to pathogen infections may originate from the limited variability and homogeneous genetic structure of *S. intermedia*. Therefore, interspecies mixing may be the only propagation mode allowing for the survival of *S. intermedia* gene pool in TNP.

If we assume that polyploid species of the *Sorbus* genus are mainly formed as a result of hybridization with the participation of diploid and polyploid species (Robertson *et al.* 2004, 2010), the rate of formation of new hybrids depends on the number and spatial distribution of parental taxa (Hamston *et al.* 2018). Since one of the parental forms (*S. intermedia*) has appeared in the Tatra Mountains relatively recently (Pusz *et al.* 2019), it can be assumed that the hybrids are at an early stage of divergence. In our study, those conclusions are supported by the observed heterogeneous levels of genetic variability between parental forms and the complex genetic structure of the population.

### The taxon of *S. carpatica*

**Figure 5.**
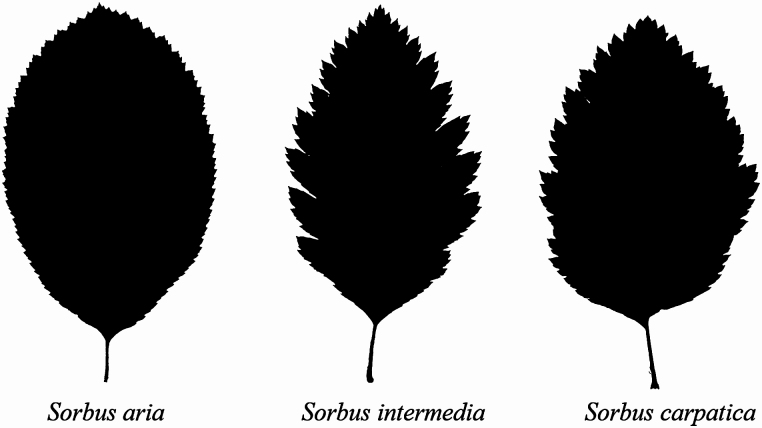
Comparison of leaves’ shapes of different *Sorbus* species.

*Sorbus carpatica* appears to be a hybrid of the diploid *S. aria* and the tetraploid *S. intermedia*. It is a tree or shrub, characterized by broad leaves on short sterile shoots. Leaves are elliptical to broadly elliptical, lobed or conspicuously double serrate (Fig. 5). However, the diversity of leaf shape observed for *S. carpatica* makes it challenging to differentiate it from other *Sorbus* species based on leaf morphology alone. Therefore, genetic analysis are needed for its proper delamination. Tested individuals of *S. carpatica* contain three alleles at some microsatellite loci, what indicates that they have a polyploidy genome and reproduce clonally. Ten identified individuals were recorded at four localities in the valleys of Tatra Mountains: Jaworzynka, Kalatówki 1, 2 and Dolina nad Capkami. However, more individuals need to be identified and studied to allow for full verification of *S. carpatica* taxon, including its genome size characteristics. Continuous research is also needed on discard contribution of other *Sorbus* species such as *S. austriaca* or *S. aucuparia* in the formation of *S. carpatica.*

## Conclusions

The process of interspecific hybridization between native and foreign species is frequently a consequence of human activity. Hybridization may lead to significant reduction or even loss of the native gene pool (Kempf *et al.* 2018). This is especially dangerous in protected areas of unique natural value, where populations occur in small areas. Our results enabled the identification of individuals from different species of the genus *Sorbus*, which participate in the hybridization process. Variation patterns at nuclear microsatellites loci allowed the distinction of a hybrid form of *S. carpatica* from the morphologically very similar *S. intermedia*. Our data provide evidence of high genetic variation and complex evolutionary history of the *S. aria*. We suggest protection of all its stands to facilitate their natural regeneration. Presented results will advance the management and design of protective strategies within the Carpathian Mountains, facilitating the identification of native, hybrid and foreign *Sorbus* species. It is important to note that the applied markers do not allow definitive determination of the ploidy level that is needed for full taxonomic identification of hybrid forms. Understanding the extent of interspecific hybridization in national parks and evaluation of the genetic diversity of parental populations is crucial for efficient conservation of genetic resources. Therefore, our work advances conservation approaches for the analysis of tree species hybridization which has to be considered when implementing protection strategies.

## Supporting Information

The following additional information is available in the online version of this article—

Table S1. Characteristics of microsatellite loci and multiplex design for PCR reaction of Sorbus genus.

Table S2. Characteristics of PCR mix and protocol. 

Table S3. Eigenvalues, the percent of variance and cumulative variance. 

Figure S1. The specific numerical distribution of the observed alleles in the studied loci for tested Sorbus genera. A1-23, C1-20, J1-30 – species adherence and number of individual (A-S. *aria, C-S. carpatica, J-S. intermedia*). SA01, SA06, SA07, MSS5, SA02, SA08, CH01h01, CH01h10, MS14h03, CH02C09, SA09, SA14, SA19.1 – locus name, 1 – 13 – locus number. Light blue rectangle - the occurrence of two alleles in the specific individual and locus, dark-blue rectangle - the occurrence of three alleles in the specific individual and locus, white rectangle – missing data. 

Figure S2. Scatterplot of the DAPC of genetic differentiation data for tested Sorbus species. The diagram showed the first two principal components of the DAPC using species adherence as prior clusters. Individuals representing different Sorbus species (after identification on the field) were shown by different colors (blue - *S. aria*, yellow - *S. carpatica*, red - *S. intermedia*). Doted lines were showing the direction of the total variance of PCA. Figure S3. The grouping of species based on principal component analysis (PCA). Abbreviations of morphological features according to Table 5, A – *Sorbus aria*, I – *Sorbus intermedia*, C – *Sorbus carpatica*. The left and bottom axes belong to the vectors of primary variables and the top and right axes belong to the scores of the samples (dots).

plaa070_suppl_Supplementary_Materials

## Data Availability

The data set and Supplementary Materials are stored in the Open Science Framework repository (https://osf.io/4j3bv/?view_only=ce6794f40dca4d56ac00e2eb96819937).

## Notes

**Evolution & Diversity. Chief Editor:** Jeremy Beaulieu
