# Supplementary material for: Hybridization and introgression of native and foreign Sorbus tree species in unique environments of protected mountainous areas
Source: AoB Plants. 2020 Dec 30;13(1):plaa070. doi: 10.1093/aobpla/plaa070 (PMC7877695; doi:10.1093/aobpla/plaa070)
Supplement: plaa070_suppl_Supplementary_Materials [file plaa070_suppl_supplementary_materials.pdf]

Supplementary materials

The data set and Supplementary Materials are stored in the Open Science Framework repository (https://osf.io/4j3bv/?view_only= ce6794f40dca4d56ac00e2eb96819937).

Study area

The Tatra Mountains are the highest mountain range in the Carpathians, with typical alpine soil and climate conditions. The geomorphological isolation and their unique climate contributed to development of vegetation characteristic only for this region (Zięba et al., 2018). The modern flora in the highest Tatra Mountains, from about 1800 m above sea level onwards, was formed during the last ice age and because of the inaccessibility of the area, it retained most of its natural character to this day. In lower mountain locations, where our plant material was collected, the Tatra nature was subjected to major human induced transformations in the last centuries, mainly due to logging of trees for developing mining, metallurgy and construction industries and to uncover land for animal grazing (Fabijanowski and Dziewolski, 1996). The area was then reforested with not only native species like Norway spruce, Silver fir or European larch but also with alien species such as Japanese larch or Swedish mountain ash, to improve the productivity of commercially used tree stands (Sokołowski, 1936; Madeyski, 1974). Emerging threats to the natural wealth of the Tatra Mountains and the awareness of the need to protect the existing ecosystem led to the establishment of the TNP in 1954. This park covers about 21 thousand ha and 70% of its area is covered by forests and dwarf pine shrubs. The remaining 30% are high mountain grasslands, rocks and water bodies. More than half of the TNP area is under strict protection, which refers to flora and fauna as well as processes taking place in the natural environment (Mirek, 1996).

Fabijanowski J, Dziewolski J. 1996. Gospodarka leśna. W: Przyroda Tatrzańskiego Parku Narodowego, Mirek Z (red.), Tatrzański Park Narodowy, Kraków-Zakopane 1996, ss. 675-696.

Madeyski S. 1974. Modrzew *Larix decidua* Mill.: Studia ośrodka dokumentacji fizjograficznej. Vol. III (ed. by S Myczkowski) Wydawnictwo Polskiej Akademii Nauk, Wrocław, Poland, pp. 71–85.

Mirek Z. 1996. Tatry i Tatrzański Park Narodowy – wiadomości ogólne: Przyroda Tatrzańskiego Par ku Narodowego (ed. by Z Mirek) Tatrzański Park Narodowy, Kraków, Zakopane, Poland, pp. 17–26.

Sokołowski S. 1936. Las Tatrzański. Wydawnictwo Popularno-Naukowe Muzeum Tatrzańskiego, Zakopane, Poland.

Zięba A, Różański W, Szwagrzyk J. 2018. Syntaxonomy of relic Swiss stone pine (*Pinus cembra*) forests in the Tatra Mountains. Tuexenia 38: 155–176. doi:10.14471/2018.38.004.

Supplementary figures and tables.

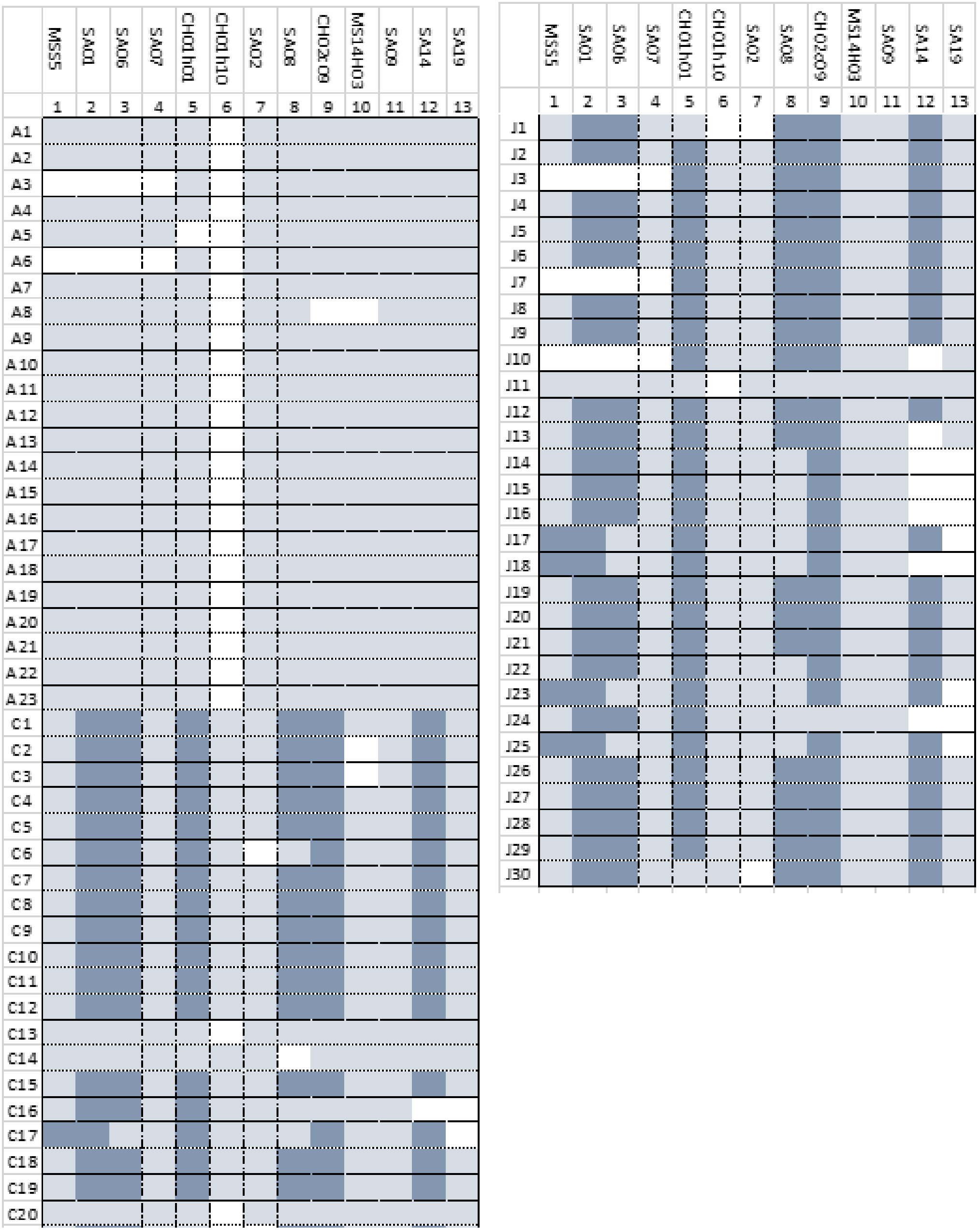

Supplementary Figure S1. The specific numerical distribution of the observed alleles in the studied loci for tested *Sorbus* genera. A1-23, C1-20, J1-30 – species adherence and number of individual (A-*S. aria*, C-*S. carpatica*, J-*S. intermedia*). SA01, SA06, SA07, MSS5, SA02, SA08, CH01h01, CH01h10, MS14h03, CH02C09, SA09, SA14, SA19.1 – locus name, 1 – 13 – locus number. Light blue rectangle - the occurrence of two alleles in the specific individual and locus, dark-blue rectangle - the occurrence of three alleles in the specific individual and locus, white rectangle – missing data.

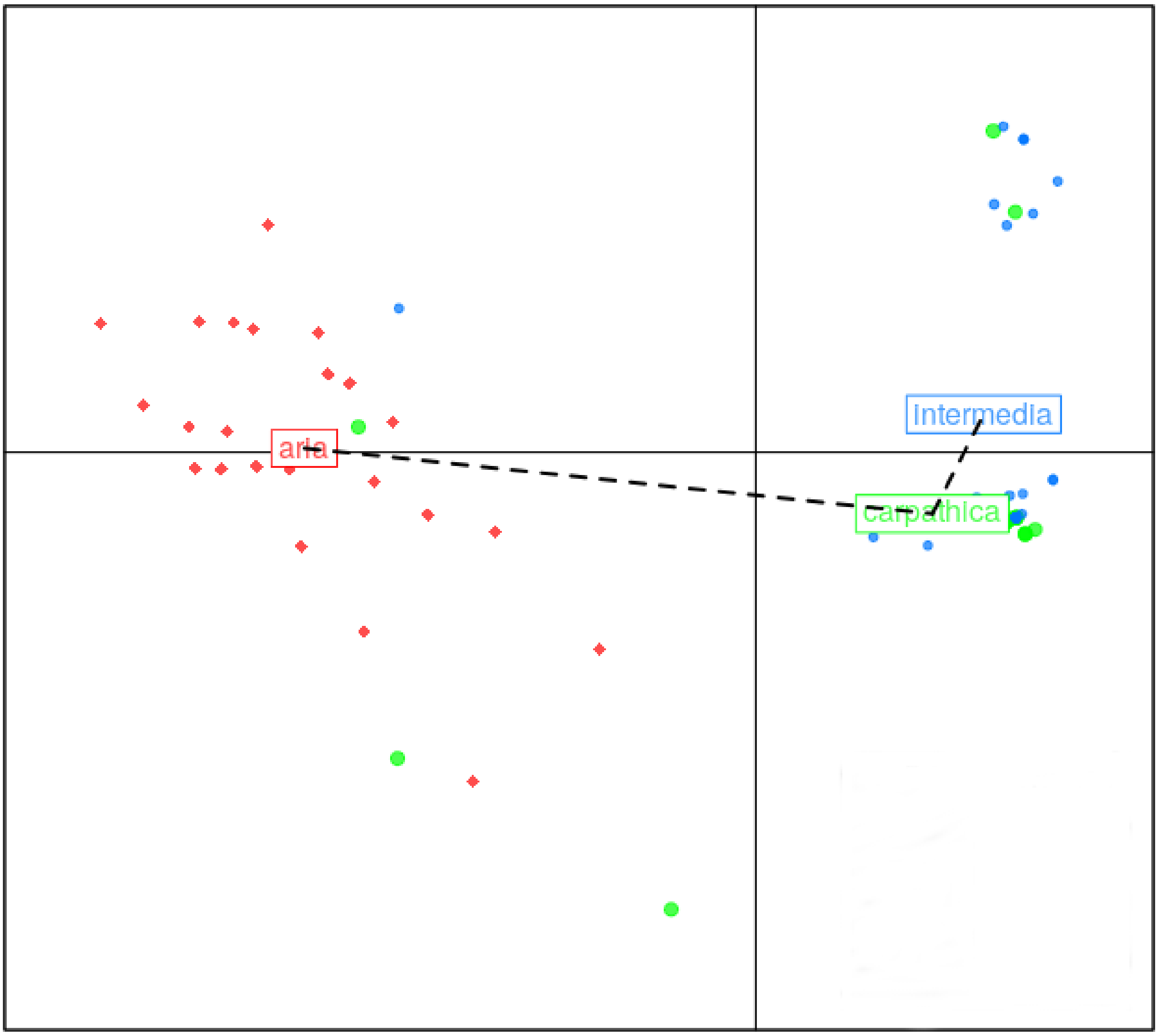

Supplementary Figure S2. Scatterplot of the DAPC of genetic differentiation data for tested *Sorbus* species. The diagram showed the first two principal components of the DAPC using species adherence as prior clusters. Individuals representing different Sorbus species (after identification on the field) were shown by different colors (blue - *S. aria*, yellow - *S. carpatica*, red - *S. intermedia*). Doted lines were showing the direction of the total variance of PCA.

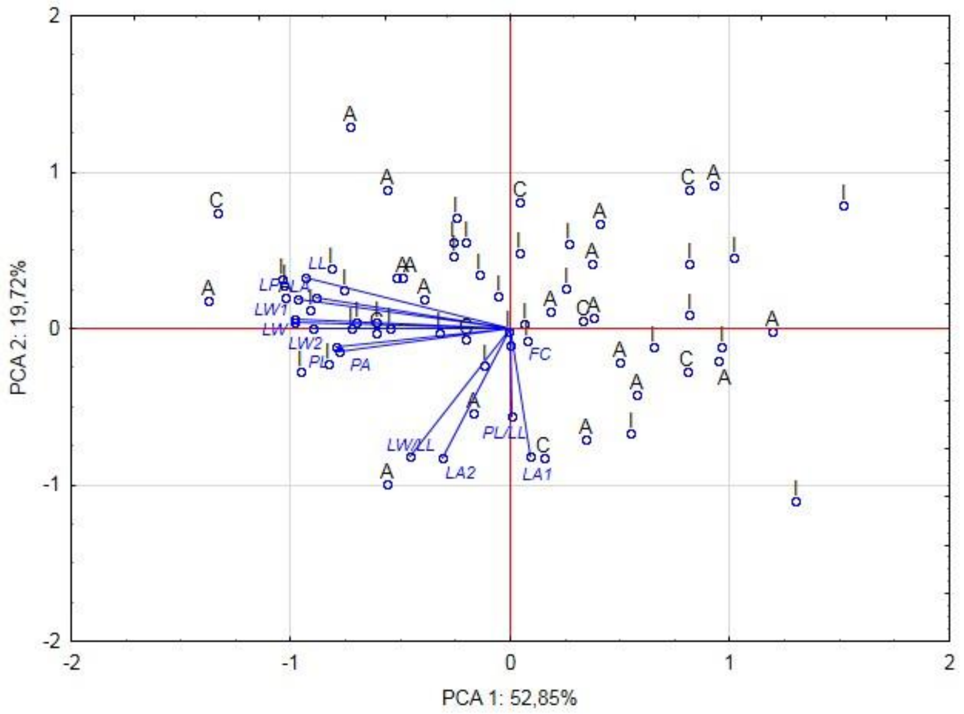

Supplementary Figure S3. The grouping of species based on principal component analysis (PCA). Abbreviations of morphological features according to Table 5, A – *Sorbus aria*, I – *Sorbus intermedia*, C – *Sorbus carpatica*. The left and bottom axes belong to the vectors of primary variables and the top and right axes belong to the scores of the samples (dots).

Supplementary Table S1. Characteristics of microsatellite loci and multiplex design for PCR reaction of Sorbus genus. * gradient PCR

No	Locus	Repeated motif	F-forward R-reverse	PCR primer sequence (5' - 3')	Multiplex	Annealing temp. (°C)	Expected length (bp)	Fluorescent dyes	Source
1.	SA01	$(GA)_{13}$	F	ATGGAGTTGAGCTCCACATC	I	60	229 (212-254)	6-FAM	González-González et al., 2010
			R	GGTGGAGGGACAATTGTGTC					
2.	SA06	$(GA)_{32}$	F	ATTTGATCCATGTGCGACTGCA	I	60	297 (248-297)	PET	González-González et al., 2010
			R	TGCAGCGGTTGCAGATTGCA					
3.	SA07	$(GA)_{15}$	F	ACGTTTTCAGTATGATGGCC	I	60	334 (325-349)	6-FAM	González-González et al., 2010
			R	CTTCGCAGTTCATTAAGCAC					
4.	MSS5	(CG)n	F	CCCCAACAACATTTTTCTCC	I	60	119-143	PET	Oddou-Muratorio et al., 2001,
			R	CCTCTCGCTCTTTGCCTCT					Kamm et al., 2009
5.	SA02	$(GA)_{16}$	F	CTAGGTATCATCTCCGACCA	II	60	293 (270-325)	NED	González-González et al., 2010
			R	ACGTAGCACTGAATGGTATAG					
6.	SA08	$(CT)_{16}$	F	CAGAGAGAGTGCACTGCCT	II	60	249 (233-287)	6-FAM	González-González et al., 2010
			R	GAATTCTTGGCAGTTTGCCT					
7.	CH01h01	(CG)N	F	GAAAGACTTGCAGTGGGAGC	II	60	93-149	PET	Gianfranceschi et al., 1998
			R	GGAGTGGGTTTGAGAAGGTT					Kamm et al., 2009
8.	CH01h10	(CG)N	F	TGCAAAGAAGGTAGATATATGCCA		55-60*	95-135	6-FAM	Gianfranceschi et al., 1998,
			R	AGGAGGGATTGTTTGTGCAC					Kamm et al., 2009
9.	MS14h03	(CG)N	F	CGCTCACCTCGTAGACGT	III	48	149-209	VIC	Liebhard et al., 2002;
			R	ATGCAATGGCTAAGCATA					Kamm et al., 2009
10.	CH02C09	(CG)N	F	TTATGTACCAACTTTGCTAACCTC	III	48	225-249	6-FAM	Liebhard et al., 2002;
			R	AGAAGCAGCAGAGGAGGATG					Kamm et al., 2009
11.	SA09	$(AG)_{17}$	F	CTTGTTGGACGGATTTCTTC	IV	55	174 (161-197)	NED	González-González et al., 2010
			R	CCAATACTTGAGTAGCATAC					
12.	SA14	$(TC)_{30}$	F	ATGGATTTAGGTTAACAGTTGTC	IV	55	203 (197-232)	PET	González-González et al., 2010
			R	GAGGTAAAACCTACCAGTATAC					
13.	SA19.1	$(GA)_{24}$	F	AAGTTTACAAGAGTGTGTTCAG	IV	55	241 (212-250)	VIC	González-González et al., 2010
			R	GAATTCATGAAAGCAGCTAATG					

Supplementary Table S2. Characteristics of PCR mix and protocol.

PCR mix	[µl]
DNA	1
Multiplex PCR Kit (Qiagen)	5
10µM primer mix	2
H_20	2
total volume	10

PCR protocol	°C/sec	
initial denaturation	95/900	
denaturation	94/30	x 35 cycles
annealing	60* or 48** or 55***/90	
elongation	72/60	
final elongation	60/1800	

* for I, ** for II and III, *** for IV multiplex, multiplex design in table S1.

For locus CH01h10 PCR reaction was carried out with use of Red Taq Ready Mix polymerase (Sigma Aldrich) and annealing with increasing temperature from 55 °C to 60 °C.

Supplementary Table S3. Eigenvalues, the percent of variance and cumulative variance.

Mean components	Eigenvalue	% Tolal Variance	Cumulative Eigenvalue	Cumulative %
1	6.87	52.85	6.87	52.85
2	2.56	19.72	9.44	72.58
3	1.69	12.99	11.12	85.57
4	1.03	7.95	12.16	93.52
5	0.40	3.11	12.56	96.62
6	0.18	1.39	12.74	98.01
7	0.11	0.84	12.85	98.85
8	0.07	0.51	12.92	99.36
9	0.04	0.33	12.96	99.69
10	0.02	0.16	12.98	99.85
11	0.01	0.08	12.99	99.94
12	0.01	0.04	13.00	99.98
13	0.01	0.02	13.00	100.00
